# Obesity-Dependent Association of the rs10454142 *PPP1R21* with Breast Cancer

**DOI:** 10.3390/biomedicines12040818

**Published:** 2024-04-08

**Authors:** Irina Ponomarenko, Konstantin Pasenov, Maria Churnosova, Inna Sorokina, Inna Aristova, Vladimir Churnosov, Marina Ponomarenko, Yuliya Reshetnikova, Evgeny Reshetnikov, Mikhail Churnosov

**Affiliations:** Department of Medical Biological Disciplines, Belgorod State National Research University, 308015 Belgorod, Russia; ponomarenko_i@bsu.edu.ru (I.P.); 944472@bsu.edu.ru (K.P.); churnosovamary@gmail.com (M.C.); sorokina@bsu.edu.ru (I.S.); aristova@bsu.edu.ru (I.A.); 958561@bsu.edu.ru (V.C.); 1256888@bsu.edu.ru (M.P.); 130226@bsu.edu.ru (Y.R.); reshetnikov@bsu.edu.ru (E.R.)

**Keywords:** *MMP*, breast cancer, obesity, SNP, association

## Abstract

The purpose of this work was to find a link between the breast cancer (BC)-risk effects of sex hormone-binding globulin (SHBG)-associated polymorphisms and obesity. The study was conducted on a sample of 1498 women (358 BC; 1140 controls) who, depending on the presence/absence of obesity, were divided into two groups: obese (119 BC; 253 controls) and non-obese (239 BC; 887 controls). Genotyping of nine SHBG-associated single nucleotide polymorphisms (SNP)—rs17496332 *PRMT6*, rs780093 *GCKR*, rs10454142 *PPP1R21*, rs3779195 *BAIAP2L1*, rs440837 *ZBTB10*, rs7910927 *JMJD1C*, rs4149056 *SLCO1B1*, rs8023580 *NR2F2*, and rs12150660 *SHBG*—was executed, and the BC-risk impact of these loci was analyzed by logistic regression separately in each group of obese/non-obese women. We found that the BC-risk effect correlated by GWAS with the SHBG-level polymorphism rs10454142 *PPP1R21* depends on the presence/absence of obesity. The SHBG-lowering allele C rs10454142 *PPP1R21* has a risk value for BC in obese women (allelic model: C*vs*T, OR = 1.52, 95%CI = 1.10–2.11, and p_perm_ = 0.013; additive model: CC*vs*TC*vs*TT, OR = 1.71, 95%CI = 1.15–2.62, and p_perm_ = 0.011; dominant model: CC + TC*vs*TT, OR = 1.95, 95%CI = 1.13–3.37, and p_perm_ = 0.017) and is not associated with the disease in women without obesity. SNP rs10454142 *PPP1R21* and 10 proxy SNPs have adipose-specific regulatory effects (epigenetic modifications of promoters/enhancers, DNA interaction with 51 transcription factors, eQTL/sQTL effects on five genes (*PPP1R21*, *RP11-460M2.1*, *GTF2A1L*, *STON1-GTF2A1L*, and *STON1*), etc.), can be “likely cancer driver” SNPs, and are involved in cancer-significant pathways. In conclusion, our study detected an obesity-dependent association of the rs10454142 *PPP1R21* with BC in women.

## 1. Introduction

BC is the most common malignant tumor in females, originating from the epithelium of the breast [1]. According to the statistical materials of the World Health Organization (WHO), the number of women in the world registered with BC is 7.8 million (2020 data), and at the same time, the number of years of healthy life lost by these women is the largest among all types of cancer [2]. No less important is the problem of the high incidence of BC for women of the Russian Federation: It ranks in the first place in both oncological pathology (22.1%) and causes of death from malignant neoplasms (15.8%) [3]. According to the forecasts of the Global Cancer Observatory WHO, among Russian women in the next 20 years (2020–2040), the number of BC cases will increase by 1.37 times, and the number of deaths from this disease will increase by 1.49 times [4].

The results of a detailed analysis of the mechanisms of the occurrence of BC, obtained by various scientific teams, indicate the involvement of hereditary factors in the occurrence of the disease—it is believed that in 31%, genetic influences determine the BC-development risk to some extent [5,6]. It has been shown that many cases of the disease (approximately in every twentieth woman) are the result of mutations in the group of so-called highly/moderately penetrant genes (*BRCA1*, *BRCA2*, *PALB2*, *CHEK2*, etc.), the presence of which in a woman’s body greatly increases the risk of BC [7,8]. The data of extensive GWAS aimed at finding the genetic causes of BC allow us to talk about more than 200 specific genetic determinants associated with the disease risk (explaining no more than 18% of the inheritance of BC) [9,10,11]. However, despite the above facts, a significant proportion of the genetic determinants of BC (more than 50% [9]) remains “hidden” and incomprehensible (the so-called “missing” heredity), requiring study for the current period of time.

In BC biology, sex hormones (estradiol, testosterone, etc.) are important, and their high concentrations cause a higher risk of the disease [12,13,14]. BC-significant biological effects of sex hormones directly depend on the content of SHBG, which binds/transports them and thus is the most important “regulator” of the level of bioavailable (so-called biologically active) forms of these sex hormones [15,16,17,18]. The concentration of circulating SHBG in the organism in more than 50% of cases is determined by hereditary factors [15,19], and by now, certain GWAS-significant loci associated with the level of SHBG are known [20]. The results obtained on the basis of Mendelian randomization (MR) demonstrate the reverse genetic relationship between the level of SHBG and the risk of BC [14,21].

A meaningful risk factor for BC development in postmenopausal women is obesity [22,23,24]: In the “excess” adipose tissue in the organism, there is an increased formation of estrogens from androgens, pro-inflammatory cytokines, insulin-like growth factors, etc., which are rather important for the BC pathophysiology [25,26,27]. In addition, the level of SHBG and SHBG-related sex hormones (androgens and estrogens) correlates significantly with BMI (obesity): Sex hormones have positive correlations with BMI (obesity) and are SHBG-negative [28,29,30,31]. Thus, it can be assumed that BMI (obesity), being a significant modifier of the level of SHBG (reduces its level) and SHBG-related sex hormones (increases their level) in the organism, can also have a prominent effect on the correlating nature of SHBG candidate genes with BC. Within the framework of this hypothesis, the present study was carried out, the purpose of which was to find an answer to the following question: “Do BC-risk effects of SHBG-related gene polymorphisms depend on obesity?”

## 2. Materials and Methods

### 2.1. Study Subjects

The study was conducted on a sample of 1498 women (358 BC; 1140 controls) who, depending on the presence/absence of obesity, were divided into two groups: obese (119 BC; 253 controls) and non-obese (239 BC; 887 controls). The presented sample sizes for the BMI ≥ 30 cohort (119 BC; 253 controls) and BMI < 30 group (239 BC; 887 controls) were sufficient to identify the following distinctions in frequencies of the genetic variants between the EnH and controls: OR = 1.56–1.95 (BMI ≥ 30) and OR = 1.36–1.56 (BMI < 30): additive model; OR = 1.73–2.10 (BMI ≥ 30) and OR = 1.48–1.64 (BMI < 30): dominant model; OR = 2.05–9.63 (BMI ≥ 30) and OR = 1.62–4.67 (BMI < 30): recessive model) (calculations were performed in the Quanto program (v. 1.2.4), and such parameters as two-sided test, with α = 0.05 and 80% power, were considered). All the necessary procedures for ethical control of the conducted research (consent to inclusion in the study from each BC/control subject, signed personally, and the support of the Medical Ethical Committee of the Belgorod State University) were carried out. BC and control samples were recruited in parallel (BC in the Belgorod Regional Oncological dispensary; control in the Belgorod Regional Clinical Hospital) for seven years (2010–2016). Histological confirmation of BC was mandatory for all cases included in the study [32]. The absence of BC, other tumors in the anamnesis, and decompensated diseases was the basis for inclusion in the control group [33,34,35]. All participants (BC/control) lived and were born in Russia (central region) and were Europeans [36,37]. The height and weight of the subjects were used in the calculation of BMI according to the standard formula [38]. The obese group included women with a BMI of 30 or more, and the group of women without obesity included subjects with a BMI less than 30 [39]. It should be noted that the BMI parameter used by us as an indicator for identifying groups of obese/non-obese women, which is based on a direct calculation taking into account height and weight, has some limitations since it does not take into account the percentage of fat in the human body; for example, a person with a high BMI having a higher the level of muscle mass may be incorrectly classified as overweight or obese. However, due to the lack of data on the waist circumference in the studied individuals, the use of which in conjunction with the BMI would eliminate the above problems, we used the BMI parameter as an approximate indicator, which allowed us to generally differentiate (but with certain limitations specified by us in the “limitation” section of this study) between individuals with obesity (BMI ≥ 30) and without obesity (BMI < 30) in the groups of women we formed.

### 2.2. SNP Selection and Genotyping

For the genetic study, DNA was isolated from the venous blood (4–5 mL of peripheral blood) of BC, and the control using the phenol-chloroform-alcohol technique was utilized [40,41]. Before carrying out the genotyping procedure on the CFX96 device (Real-Time PCR System (Bio-Rad Laboratories, Inc., Hercules, CA, USA)) [42], all DNA samples were tested on a spectrophotometer Nanodrop-2000 (Thermo Fisher Scientific Inc., Waltham, MA, USA), with which their purity and concentration were evaluated [43,44].

Genotyping of nine SHBG associated previously by GWAS with SNPs (Table S1) [20,45,46,47,48,49] (rs17496332 *PRMT6*, rs780093 *GCKR*, rs10454142 *PPP1R21*, rs3779195 *BAIAP2L1*, rs440837 *ZBTB10*, rs7910927 *JMJD1C*, rs4149056 *SLCO1B1*, rs8023580 *NR2F2*, and rs12150660 *SHBG*) was executed. To assess the quality of the obtained genetic experimental data, a well-established procedure of “blind” re-genotyping was used [50], as a result of which there was a complete “coincidence” of 99% of the data of primary and repeated genotyping.

### 2.3. Statistical, Bioinformatics Analysis

In both studied cohorts (obese and non-obese women), the actual distribution of genetic variants (genotypes) in BC and controls was analyzed for compliance with what was expected when implementing the Hardy–Weinberg law [51]. The BC-risk impact of the examined nine SHBG-impact loci was analyzed by logistic regression separately in each group of obese/non-obese women. Four well-renowned genetic models, namely additive, recessive, dominant, and allelic, were considered [52,53]. Covariates (age/BMI [54]) and permutations (in order to minimize false-positive results [55,56]) were used in computations of the values’ odds ratio (OR) and their confidence intervals (95%CI) [57] in the gPLINK program [58]. As an indicator “denoting” reliable data, we adopted p_perm_ < 0.025. (The Bonferroni correction was imposed equal to 2 based on the number of pairs being compared, with and without obesity [59]). The power of the identified SNP–BC associative links was evaluated in the Quanto program (v. 1.2.4) [60].

The possible functionality of the BC-correlated locus rs10454142 *PPP1R21* and LD SNPs were discovered by in silico tactics [61,62,63] and several bioinformatics databases/resources [64,65], such as QTLbase (accessed on 30 August 2023) [66], regBase-CAN (accessed on 30 August 2023) [67], HaploReg (accessed on 05 August 2023) [68], GTExportal (accessed on 10 August 2023) [69], Gene Ontology (accessed on 20 August 2023) [70], and STRING (accessed on 20 August 2023) [71].

## 3. Results

Table 1 presents the BC-significant biological/biochemical/clinicopathological parameters of the participants in this study. We presented them earlier in the previous genetic work performed on the same samples of BC/control with obesity/without obesity [39]. BC patients, both among individuals with a BMI ≥ 30 and among individuals with a BMI < 30, had a higher BMI (34.95 and 27.55, respectively) compared to the control (33.12 *p* = 0.001 and 26.13 *p* = 0.0004, respectively). Based on this, the BMI and the age of the woman were included in the genetic calculations as confounders [39].

In non-obese (Table S2) and obese (Table S3) women, the actual distribution of genetic variants (genotypes) fully corresponded with that expected when the Hardy–Weinberg law was fulfilled (*p* > 0.05).

We found that the BC-risk effect correlated by GWAS with the SHBG-level polymorphism rs10454142 *PPP1R21* depends on the presence/absence of obesity (Table 2). The allele C rs10454142 *PPP1R21* has a risk value for BC in obese women (allelic model: C*vs*T, OR = 1.52, 95%CI = 1.10–2.11, *p* = 0.012, and p_perm_ = 0.013; additive model: CC*vs*TC*vs*TT, OR = 1.71, 95%CI = 1.15–2.62, *p* = 0.009, p_perm_ = 0.011, and power = 92.16%); dominant model: CC + TC*vs*TT, OR = 1.95, 95%CI = 1.13–3.37, *p* = 0.016, p_perm_ = 0.017, and power = 82.98%) and is not associated with the disease in the cohort of women without obesity (Table 2).

### 3.1. Possible Functionality of the BC-Correlated Locus rs10454142 PPP1R21 (In Silico Data)

In this section of the work, we evaluate the possible functional value of rs10454142 *PPP1R21* and 10 strongly coupled SNPs (r ≥ 0.80) in the following: (a) the liver (the main organ of SHBG synthesis in the organism [16]), (b) adipose tissue (the obesity-dependent association of the rs10454142 *PPP1R21* with BC we found in this work), and (c) in general in the organism.

#### 3.1.1. Liver-Specific Regulatory Effects of BC-Causal Loci

It was revealed that rs10454142 *PPP1R21* (position—48419260) and two adjacent (in adjacent positions of nucleotides—48419259 and 48419261) and strongly linked SNPs (r^2^ = 1.00 and LD = 1.00), namely rs201414717 and rs10454143, are epigenetic modifiers of gene activity in the liver (Haploreg data, accessed 20 August 2023): These SNPs were localized in regions of histone proteins-labelling enhancers (H3K4me1), promoters (H3K4me3), active enhancers (H3K27ac), and active promoters (H3K9ac) in hepatocytes (Table 3). According to the materials of the QTLbase database (accessed 20 August 2023), rs10454142 *PPP1R21* and 10 loci in linkage disequilibrium (LD) with it were linked to the level of genome methylation (cg15846641) in the liver (liver hepatocellular carcinoma) (Table S4), and the BC-risk allele C rs10454142 *PPP1R21* was associated with hypermethylation of the above DNA site (beta = 0.46 *p* = 4.31 × 10^−9^), which may lead to a decrease in gene expression. Interestingly, the genetic variant C rs10454142 *PPP1R21* is associated with low transcription of the *GTF2A1L* gene in the liver (NES = −0.47; *p* = 0.0000012); the expression of this gene in the liver is also affected by eight strongly linked SNPs (Table 3 and Table S5). 

#### 3.1.2. Adipose-Specific Regulatory Effects of BC-Causal Loci

The epigenetic information presented in the Haploreg database indicates a serious role in the modification of the gene activity of rs10454142 *PPP1R21* and two proxy loci (rs201414717 and rs10454143) due to the location in enhancers, active promoters, and enhancers active in adipose tissue (mesenchymal stem-cell-derived adipocyte-cultured cells and adipose nuclei) (Table 3).

GTE-portal materials show the effect of rs10454142 *PPP1R21* on gene expression in both visceral and subcutaneous adipose tissue (Table 3 and Table S5). The allelic variant C rs10454142, which is a risk factor for BC, is associated with reduced transcription of three genes, namely *GTF2A1L* (NES = −0.46; *p* = 1.68 × 10^−11^), *RP11-460M2.1* (NES = −0.39; *p* = 1.8 × 10^−8^), and *STON1-GTF2A1L* (NES = −0.37; *p* = 7.2 × 10^−8^), in visceral adipose tissue and two genes, namely *GTF2A1L* (NES = −0.55; *p* = 2.1 × 10^−22^) and *STON1-GTF2A1L* (NES = −0.36; *p* = 1.7 × 10^−9^), in subcutaneous adipose tissue. This genetic variant is also associated with the level of splicing of four genes (*GTF2A1L*, *STON1*, *PPP1R21*, and *STON1-GTF2A1L*) in visceral and three genes (*GTF2A1L*, *STON1*, and *PPP1R21*) in subcutaneous adipose tissue (Table 3 and Table S6).

Moreover, the C allele marks a reduced level of *PPP1R21* gene splicing in visceral (IntronID:48505857:48507269:clu_47092; NES = −0.37; *p* = 1.3 × 10^−10^) as well as in subcutaneous (IntronID:48505857:48507269:clu_48393; NES = −0.41; *p* = 8.7 × 10^−12^) adipose tissue and increased values of alternative splicing of *GTF2A1L* (IntronID:48591855:48595228:clu_47096; NES = 0.49; *p* = 1.1 × 10^−10^), *STON1* (IntronID:48591855:48595228:clu_47096; NES = 0.49; *p* = 1.1 × 10^−10^), and *STON1-GTF2A1L* (IntronID:48769074:48776280:clu_47101; NES = 0.32; *p* = 0.0000025) in visceral adipose tissue and *GTF2A1L* (IntronID:48591855:48595228:clu_48397; NES = 0.50; *p* = 5.5 × 10^−14^) and *STON1* (IntronID:48591855:48595228:clu_48397; NES = 0.50; *p* = 5.5 × 10^−14^) genes in subcutaneous adipose tissue.

The foregoing data indicate, firstly, significant eQTL/sQTL effects in the fat tissue of BC-involved SNP rs10454142 in relation to five genes (*RP11-460M2.1*, *GTF2A1L*, *STON1-GTF2A1L*, *STON1*, and *PPP1R21*). Secondly, eQTL/sQTL effects of the BC-risk allele variant C rs10454142 *PPP1R21* in relation to individual genes in the visceral and subcutaneous adipose tissue were unidirectional. (This allele is associated with increased/decreased expression/splicing of the same genes in both visceral and subcutaneous adipose tissue).

In addition, we established pronounced eQTL (three genes—*GTF2A1L*, *STON1-GTF2A1L*, and *RP11-460M2.1*) and sQTL (four genes—*GTF2A1L*, *STON1*, *PPP1R21*, and *STON1-GTF2A1L*) effects in visceral and subcutaneous adipose tissue of eight polymorphic loci highly linked with rs10454142 *PPP1R21* (Table 3, Tables S5 and S6). Thus, eight LD SNPs are now also eQTL/sQTL-significant in the adipose tissue in relation to the five genes *RP11-460M2.1*, *GTF2A1L*, *STON1-GTF2A1L*, *STON1*, and *PPP1R21*.

#### 3.1.3. Organism-Significant Regulatory Effects of BC-Causal Loci

**Figure 1:**
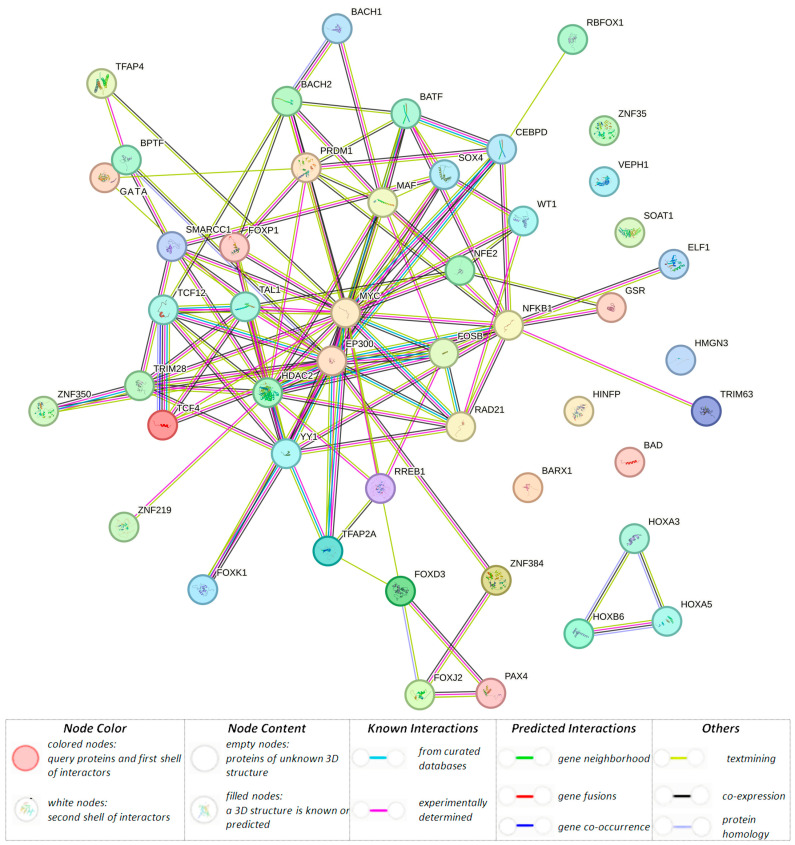
Network of interaction of transcription factors associated with BC risk, mediated by rs10454142 *PPP1R21* and proxy SNPs (STRING data).

Pursuant to in silico materials (data HaploReg v.4.2), rs10454142 *PPP1R21* and eight LD variants were located in the position of DNA motifs interacting with a variety of transcription factors (TFs) (n = 51) (Table 3 and Table S7). Using the STRING program, we analyzed the interaction of these 51 BC-associated TFs. The network of interactions obtained as a result of this analysis is shown in Figure 1. The primary role (score ≥ 0.990) in the formation of this network of paired interactions, namely YY1–EP300, TCF12–TAL1, MYC–EP300, YY1–HDAC2, NFKB1–EP300, MAF–BACH2, and TCF4–TCF12, should be noted. According to the STRING materials, interactions were carried out through protein domains (SMART data) of the basic region leucin zipper (FOSB, BACH2, MAF, BACH1, CEBPD, and NFE2) (SM00338; p(FRD) = 2.90 × 10^−6^)), helix-loop-helix domain (TFAP4, TAL1, TCF4, TCF12, and MYC) (SM00353; p(FRD) = 0.0028)), zinc finger (ZNF350, YY1, HINFP, ZNF219, PRDM1, WT1, RREB1, ZNF35, and ZNF384) (SM00355; p(FRD) =0.0320), bromo domain (TRIM28, EP300, and BPTF) (SM00297; p(FRD) = 0.0390), and FORKHEAD (FOXJ2, FOXK1, and FOXD3) (SM00339; p(FRD) = 0.0476) due to (according to local network STRING data) transcription coregulator binding. Additionally, activation of the TFAP2 (AP-2) family of transcription factors (BACH2, EP300, BATF, TAL1, MAF, PRDM1, FOXP1, and TFAP2A) (CL:20714; p(FRD) = 5.31 × 10^−6^), and T-helper 17 cell lineage commitment as well as regulation of TP53 expression (BACH2, BATF, MAF, and PRDM1) (CL:20735; p(FRD) = 8.78 × 10^−6^), granulocyte differentiation, and RUNX3 regulate the p14-ARF (BACH2, BATF, TAL1, MAF, and PRDM1) (CL:20720; p(FRD) = 3.39 × 10^−6^), homeobox, conserved site, and domain first found in the mice T locus (Brachyury) protein (HOXA5, BARX1, FOXD3, HOXB6, and HOXA3) (CL:20257; p(FRD) = 0.0063), homeobox protein, antennapedia type, conserved site, and proximal/distal pattern formation (HOXA5, HOXB6, HOXA3) (CL:20390; p(FRD) = 0.0377)).

The various biological pathways (mainly tumor-related) identified by us on the basis of information from the KEGG Pathways database are very interesting, in which the 51 transcription factors under consideration are involved: microRNAs in cancer (NFKB1, SOX4, EP300, HDAC2, and MYC) (hsa05206; p(FRD) = 0.0136), chronic myeloid leukemia (NFKB1, BAD, HDAC2, and MYC) (hsa05220; p(FRD) = 0.0136), cell cycle (EP300, RAD21, HDAC2, and MYC) (hsa04110; p(FRD) = 0.0261), thyroid hormone signaling pathway (EP300, BAD, HDAC2, and MYC) (hsa04919; p(FRD) = 0.0261), hepatitis B (NFKB1, EP300, BAD, and MYC) (hsa05161; p(FRD) = 0.0431), transcriptional misregulation in cancer (NFKB1, MAF, HDAC2, and MYC) (hsa05202; p(FRD) = 0.0431), acute myeloid leukemia (NFKB1, BAD, and MYC) (hsa05221; p(FRD) = 0.0431), and viral carcinogenesis (NFKB1, EP300, BAD, and HDAC2) (hsa05203; p(FRD) = 0.0460).

The SNP rs10454142 *PPP1R21* and 10 proxy loci were found to be located in the region of the three genes *PPP1R21*, *FOXN2*, and *KLRAQ1* and are important epigenetic modifiers for them (Table S7). The studied polymorphisms affect the level of methylation of various genome sites (QTLbase materials, accessed 20 August 2023) in blood (cg07289618, cg06092244, cg14965639, cg01450842, etc.), immunocompetent cells (CD14+ monocytes (cg14698961, cg14672293, cg14965639, cg04934807, etc.), naive T-cells CD4+ (cg12300939), and the brain’s cortex (cg15577373) (Table S4). It was revealed that the SNP rs10454142 *PPP1R2*1 and eight LD SNPs (data on two loci, rs201414717 and rs4638844, were not presented in the GTE-portal database) in various organs of the organism affect the expression of ten genes (*FOXN2*, *GTF2A1L*, *LHCGR*, *MSH6*, *PPP1R21*, *RP11-191L17.1RP11-460M2.1*, *FSHR*, *STON1*, and *STON1-GTF2A1L*) (Table S5) and alternative splicing of four genes (*GTF2A1L*, *PPP1R21*, *STON1*, and *STON1-GTF2A1L*) (Table S6). So, the BC-causal SNP rs10454142 *PPP1R21* and 10 SNPs strongly linked to it are functionally weighty in relation to 11 genes (*FOXN2*, *GTF2A1L*, *LHCGR*, *MSH6*, *RP11-191L17.1*, *KLRAQ1*, *RP11-460M2.1*, *FSHR*, *PPP1R21*, *STON1-GTF2A1L*, and *STON1*).

**Figure 2:**
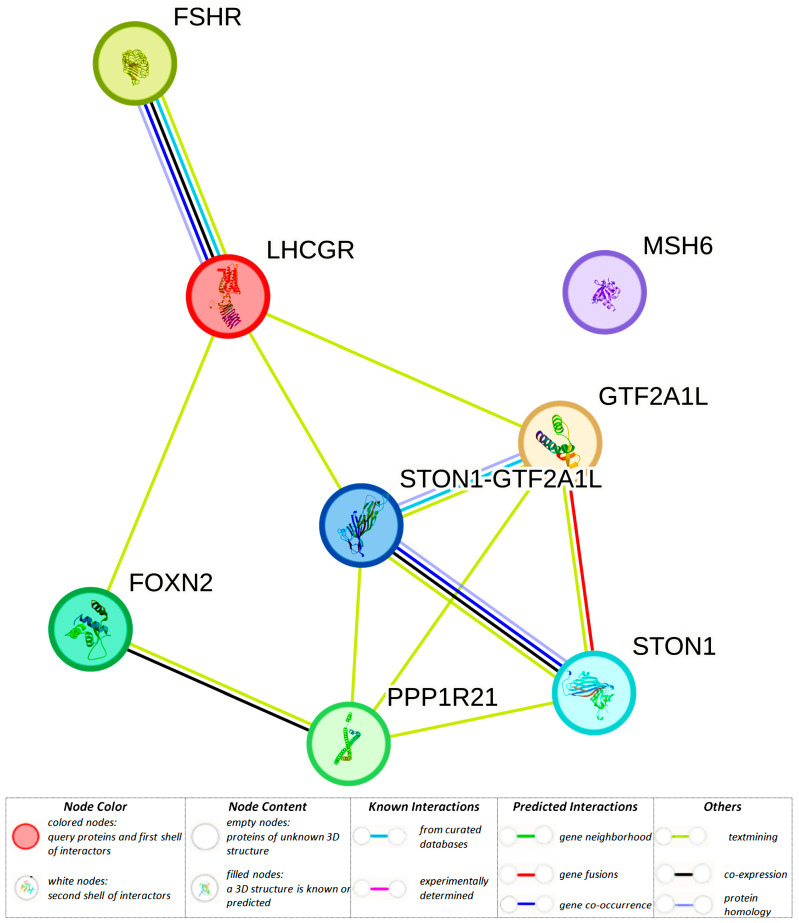
A network of protein interactions involved in the BC development due to candidate genes functionally related to rs10454142 *PPP1R21* and proxy SNP (STRING data).

Using the STRING program, we studied the interactions of proteins controlled by the aforementioned genes (Figure 2). The most pronounced cooperations were found for the protein products of the *STON1-GTF2A1L*, *STON1,* and *GTF2A1L* genes as well as *LHCGR* and *FSHR* (Figure 2). It was revealed that these communications (local network STRING data) were based on the effects of transcription factor IIA, alpha/beta subunit, and stonin (PPP1R21, FOXN2, STON1-GTF2A1L, GTF2A1L, and STON1) (CL:27077; p(FRD) = 1.20 × 10^−10^) and hormone ligand-binding receptors (LHCGR and FSHR) (CL:24198; p(FRD) = 0.0171), and these interactions are carried out with the participation of protein domains (InterPro data) of the glycoprotein hormone receptor family (LHCGR and FSHR) (IPR002131; p(FRD) = 0.0104), transcription factor IIA, alpha/beta subunit (STON1-GTF2A1L, and GTF2A1L) (IPR004855; p(FRD) = 0.0104), transcription factor IIA, beta-barrel (STON1-GTF2A1L and GTF2A1L) (IPR009088; p(FRD) = 0.0104), stonin homology (STON1-GTF2A1L and STON1) (IPR012320; p(FRD) = 0.0104), BspA-type leucine-rich repeat region (LHCGR and FSHR) (IPR026906; p(FRD) = 0.0136), AP-2 complex subunit mu, C-terminal superfamily (STON1-GTF2A1L and STON1) (IPR036168; p(FRD) = 0.0157), and Mu homology domain (STON1-GTF2A1L, and STON1) (IPR028565; p(FRD) = 0.0177).

Taking into account the fact of the presence of the main effect of rs10454142 *PPP1R21* in the occurrence of BC, we analyzed in detail the potential association with the tumors development of this polymorphism and 10 strongly linked SNPs (Table 4) using the regBase-CAN database. It was found that eight out of the ten loci considered (materials on the rs201414717 locus were not presented in the regBase-CAN database) are the most likely drivers of the occurrence of tumors (“likely cancer driver”). These materials indicate the most important role of this genome region in the formation of tumors and are fully consistent with the data we obtained on its involvement in the development of BC.

## 4. Discussion

In this work, it was established for the first time that the BC-risk effect correlated by GWAS with the SHBG-level polymorphism depends on the presence/absence of obesity: The SHBG-lowering allele C rs10454142 *PPP1R21* has a risk value for BC in obese women (OR = 1.52–1.95) and is not associated with the disease in women without obesity.

The allele variant C rs10454142 *PPP1R21*, which, according to the results of our work, increases the risk of developing BC by 52–95% in obese women, is, according to GWAS results found by Coviello et al., associated with a low concentration of circulating SHBG [20]. Using the MR method by Dimou et al., a genetic link was found between a high level of SHBG and a low risk of BC in general (OR = 0.94) and an ER-positive variant of the tumor (OR = 0.92) but a high risk of an ER-negative form of the disease (OR = 1.09) in postmenopausal women [21]. In the work of Chen al., also using the MR procedure, a reverse genetic relationship between the level of SHBG and ER-positive BC was shown but also a direct correlation between the SHBG level and ER-negative BC [14]. It should be noted that the sample of patients studied by us predominantly has ER-positive BC (69% in patients with BMI ≥ 30), which is fully consistent with the above literature materials on this theme.

SHBG, due to the presence of steroid-interacting sites, binds/transports testosterone, estradiol, and other sex steroids in plasma, thus affecting their bioavailability [72]. Inverse correlations have been shown between the content of circulating SHBG and the concentration of bioavailable testosterone and estrogens in a woman’s body [15,16,17,18]. Thus, women with low levels of SHBG (caused, among other things, by genetic factors, for example, the allele with rs10454142 *PPP1R21*, etc.) will have an increased level of free (active) testosterone and estrogens, the risk value of which for BC has been shown in numerous previous studies [13,73,74,75,76].

It is well known that the main place formation of SHBG in the organism is the liver [16]. Based on this, it can be assumed that possible biological mechanism underlying the known GWAS connection of rs10454142 *PPP1R21* with the level of circulating SHBG in the organism [20] could be the significant functional effects of rs10454142 *PPP1R21* and the loci strongly linked to the liver, as established by us in silico (localized in the regulatory regions of the genome (promoters/active promoters and enhancers/active enhancers) and correlating with the level of DNA methylation and transcription of the *GTF2A1L* gene). The *GTF2A1L* gene encodes one of the subunits (similar subunit 1) of the general TF IIA, which has a “key” importance in the regulation of gene expression. (This subunit is specific for germ cells [77]). This TF participates in the interaction of the TATA (promoter)-binding protein and DNA (the promoter region of the gene “TATA” box), which provides the assembly and stability of the “RNA polymerase–promoter” complex necessary for the start of transcription (the pre-initiation stage of transcription) [78]. The *GTF2A1L* gene is cancer-significant, and there are materials on the relationship of its expression with such oncological diseases as breast angiosarcoma [79], endometrial carcinoma [80], bladder cancer [81], and medulloblastoma in children [82].

The link of BMI (as characterizing the content of adipose tissue in the organism) with risk of BC has been known for a long time [22,23,24,25,26,27,83,84,85]. It is believed that a high BMI represents a high risk value for BC in postmenopausal women (due to higher production of estrogens from androgens under the influence of increased aromatase activity associated with a significant fat content in the organism; increased production of pro-inflammatory cytokines; development of hyperinsulinemia and insulin resistance, leading to hyper-production of insulin-like growth factors; etc. [25,26,27]) and a protective effect in premenopausal women: With a high fat content in the organism, a longer anovulatory cycle is observed, which causes lower concentrations of progesterone and estrogens [24,84,85]. It should be emphasized that the established risk value of obesity for BC established by us in the studied cohort of women mainly (two-thirds of the group) of postmenopausal age (OR = 1.74) is fully consistent with the above literature materials on this topic [24,84].

The differences in the associations nature of rs10454142 *PPP1R21*, correlated with the SHBG level in the organism, with BC in women, depending on the obesity presence/absence, may be based on the following mechanisms. Firstly, according to the literature, the level of SHBG and SHBG-related sex hormones (androgens and estrogens) in postmenopausal women is significantly correlated with BMI [28,29,30,86,87,88,89]. Numerous clinical and experimental studies have convincingly shown that in postmenopausal women, BMI (obesity) is positively associated with both estrogens and androgens (including free ones) and negatively with SHBG [28,29,30]. Interestingly, a significant weight loss in postmenopausal women led to a significant decrease in estrogens (estrone and estradiol), free estradiol, and free testosterone and an increase in the content of circulating SHBG [86,87,88], while an increase in BMI in women of this age cohort caused a marked increase in the concentration of estrone (by 21–34%), estradiol (45–68%), free estradiol (101%), and free testosterone (35%) and a decrease in the level of SHBG (by 29–35%) [89], and these changes in the concentrations of SHBG and SHBG-related sex hormones are of paramount importance for the risk of BC in postmenopausal women (reduced and increased risk, respectively) [90]. The MR method shows “causal” negative associations between increased BMI and low SHBG levels [31].

Therefore, in postmenopausal women with obesity characterized, according to the literature, by low SHBG and a high content of SHBG-bound sex hormones (estrogen and testosterone, including their bioavailable forms) [28,29,30,31], the SHBG-lowering allelic variant C rs10454142 *PPP1R21* [20] apparently led to a more pronounced decrease in the level of BC-protective circulating SHBG in the organism [13,14,21,75,91] and, accordingly, to a more significant increase in BC-risk SHBG-related sex hormones (including their free forms) such as estrogens [17,25,74,75,92] and testosterone [13,15,18,76,93], which ultimately, according to our data, caused a high risk of BC developing in obese women (OR = 1.52–1.95), whereas in women without obesity, we did not register the association of this polymorphism with the disease (*p* > 0.30).

Secondly, according to our in silico data, rs10454142 *PPP1R21* and proxy loci are functionally significant in adipose tissue (located in enhancers and active promoters/enhancers and are eQTL/sQTL-significant in relation to the five genes *STON1*, *STON1-GTF2A1L*, *RP11-460M2.1*, *GTF2A1L*, and *PPP1R21*). Moreover, the BC-risk allele C rs10454142 was associated with a lower level of three genes’ expression (*STON1-GTF2A1L*, *GTF2A1L*, and *RP11-460M2.1*) and splicing of the *PPP1R21* gene in adipose tissue, and also, along with this, this allele has been associated with a higher level of three genes’ splicing, namely *STON1*, *STON1-GTF2A1L*, and *GTF2A1L*, in adipose tissue.

The *STON1* gene encodes the protein stonin 1, which is an important component of the endocytic apparatus and thereby participates in the molecular mechanisms of the endocytosis of cell surface proteins [94]. It is believed that stonin 1, participating in the processes of local adhesion on the cell surface and being a specific adapter of oncogenic proteoglycan neuron-glial antigen 2 (serves as a co-receptor of integrins and platelet growth factor receptor), can modulate the mobility of tumor cells and thereby promotes tumor growth [95,96]. It has been experimentally proven that in the absence of stonin 1, neuron-glial antigen 2 accumulates on the cell surface, which leads to “activation” of cell migration, and conversely, the presence of stonin 1 improves internalization of neuron-glial antigen 2 (due to stonin 1-mediated endocytosis of this oncogenic proteoglycan), which disrupts the “work” of the system’s local adhesion and leads to a decrease in the level of cellular signaling of this system and a decrease in cell mobility (including tumor cells) [95].

The results of numerous epidemiological and bioinformatic studies convincingly show the connection of the *STON1* gene (its expression) with BC (on a model of BC-specific cell lines (MCF-7)) [97,98,99] as well as the same with other various oncological diseases: pancreatic duct adenocarcinoma [100], lung cancer [101,102,103], colorectal cancer [104,105,106], papillary renal cell carcinoma [107], colon carcinoma (liver metastases) [108], basal cell skin cancer [109], clear cell kidney carcinoma [96], bladder carcinoma [110,111], and glioma [112], including in the GWAS data (lung cancer [103]). It is important to emphasize that, according to our in silico data (obtained from the regBase-CAN database), the BC-associated locus rs201414717 *PPP1R21* considered in this work and the seven SNPs strongly linked to them are the most likely drivers of the occurrence of tumors (“likely cancer driver”).

According to the information specified in the GeneCards database [113], in a number of rare cases, as a result of simultaneous transcription of nearby *STON1* and *GTF2A1L* genes, a “fused” *STON1-GTF2A1L* mRNA is formed, which subsequently undergoes alternative splicing, which leads to the formation of various protein variants consisting of separate “elements” of stonin 1 proteins and a common TF IIA (a similar subunit 1), the function of which remains unexplored to date. A number of studies have shown the association of *STON1-GTF2A1L* RNA expression with such oncological diseases as ovarian cancer [114], cervical cancer [115], stomach [116,117], and colorectal cancer [104].

The literature indicates that the genome district in the rs10454142 *PPP1R21* region (genes *STON1*, *STON1-GTF2A1L*, *GTF2A1L*, etc.) has been potentially associated with adipocyte metabolism [118,119,120,121]. Cao C.H. et al. carried out a deep, complex genetic functional analysis (3D genome interactions based on high-throughput chromosome conformation capture technology (Hi-C), eQTL, RNA-Seq, DNase-Seq, ChIP-Seq, and sing-cell sequencing) proving the relationship of *STON1*-coexpressed genes (*PPP1R21*, *LHCGR*, *FOXN2*, *STON1-GTF2A1L*, *GTF2A1L*, etc.) with metabolic processes in adipocytes (*p* = 0.0001), which was confirmed in the adipose tissue (*p* < 0.0001) and ovaries (*p* = 0.0035) of mice fed on fats [119]. The authors also revealed pronounced, multidirectional, sex-specific correlations between the expression of *STON1* and BMI in male and female adipocyte tissue: In male adipocytes, BMI positively correlated with the *STON1* expression, but in female adipocytes, these correlations were negative [119]. The features of circulating *STON1-GTF2A1L* RNA expression in epicardial adipose tissue in individuals with/without heart failure were shown in the work of He S. et al. [121]. Experimental and bioinformatic studies have demonstrated that the region of the genome in the district of the *STON1-GTF2A1L* and *GTF2A1L* genes is targeted in the processes of fructose-dependent changes in gene methylation in adipocytes and has been associated with the transformation of morphologically differentiating adipocytes into more mature and metabolically stable ones [118]. Distinctions in the differential expression of the *GTF2A1L* gene (hypoexpression) when comparing metabolically “unhealthy” obese patients and healthy obese individuals were found by Prashanth et al. [120].

The results of a sufficiently large number of GWAS indicate associations of SNPs located in the area of the *STON1-GTF2A1L*, *GTF2A1L*, and *STON1* genes with such BC-significant BMI-related signs as height [122], weight [123], waist circumference [124], waist-to-hip ratio [125,126,127,128,129], gluteal-femoral adipose tissue volume [130], body shape index [128], BMI [123,126,127,129,131,132,133,134], and body size in adults [135].

It seems important that this genome “territory” was associated by GWAS with such BC/hormone/adipocyte/BMI-significant signs as the age of menopause in women [136], lipid profile (triglycerides, HDL, and LDL) [123,133,137,138,139,140,141], as well as with adipocyte-, hormone-, and BMI-significant disease characterized by hyperandrogenic status, such as polycystic ovaries [142,143]. Cao C.H. et al., in an experiment with mice that served as a model for polycystic ovaries, revealed the link of such a TF as androgen receptors with the level of expression of the *STON1* gene in the ovaries of experimental animals [119]. The authors showed that in patients with polycystic ovaries, high expression of *STON1* may be responsible for the hyperandrogenic phenotype associated with severe metabolic disorders [119]. Differences in the levels of expression (hyperexpression) and methylation (hypomethylation) of the *STON1-GTF2A1L* gene in patients with polycystic ovaries were also demonstrated in the work of Jones M.R. et al. [144]. Polymorphisms, localized in the regions of the *STON1-GTF2A1L*, *GTF2A1L*, and *STON1* genes, according to the GWAS results, have been also associated with testosterone levels [47,48].

A number of previous studies have also shown the correlation between BMI, *SHBG* polymorphism, and BC [145,146]. Interesting data on the modifying effect of BMI on the relationship of *SHBG* polymorphism with BC were obtained in the study of Cui Y. et al. [145]. The authors, having studied a sample of 1106 patients with BC and 1180 controls (Shanghai population of China), showed that the SNP rs6259 (Asp327Asn) *SHBG* had the most pronounced associations with BC in women with low BMI (OR = 0.46) as well as in individuals with an ER-positive form of the disease (OR = 0.64), whereas in patients with ER-negative disorder, this polymorphism was not associated with the BC risk [145]. This work also demonstrated a significant effect of rs6259 on the level of SHBG in healthy postmenopausal women, especially those with low BMI: The concentration of SHBG in individuals with the Asn allele in the genotype was 10% higher than that of women without this allele (the genotype contained two Asp alleles), and at the same time, in women with low BMI, these differences in SHBG content were 20% [145]. The “protective” effect of rs6259 (Asp327Asn) in the Asian population was also shown in the work of Zhang B. et al. in which a sample of 1144 patients and 1256 controls of the Shanghai Breast Cancer Study was analyzed, and the most pronounced associations of this SNP with BC were recorded in lean (BMI < 23), postmenopausal women [146]. Interestingly, in our earlier study (the same sample of patients/controls was studied), the modifying effect of obesity on the correlation of the functionally significant SNPs of matrix metalloproteinases genes with BC risk was shown; at that, in obese women, c.836 A > G *MMP9* (rs17576) and c.1721 C > G *MMP9* (rs2250889) were disease-associated, and in non-obese women, c.-1306 C > T *MMP2* (rs243865) and c.1331-163 G > A *MMP9* (rs3787268) were BC-linked [39].

Interestingly, along with obesity, which, according to our data, is a significant factor- modifier of SNP–BC connection, other factors affecting the risk of developing BC (hormonal therapy, reproductive history, etc.) and lifestyle factors related to obesity (dietary habits, physical activity, etc.) may be potentially significant as modifiers for the realization of the phenotypic effects of BC-predisposition genes [147,148,149,150,151]. It is obvious that hormone therapy (estrogen/progestogen), which is often prescribed to women in the menopausal period [147], will have a significant effect on the hormonal status of the organism (including the content of sex and other hormones) and due to this can modify the SNP_BC interactions. The McTiernan et al. review paper provides convincing evidence of an association of the highest level of physical activity (comparisons were made with the lowest levels of physical activity) with a reduced risk of developing BC and other forms of cancer [148]. At the same time, the indicators of relative risk reduction for BC amounted to 12–21% [148]. The most important modifiers of breast cancer risk and, accordingly, SNP–breast cancer interaction may be reproductive factors (age at menarche and menopause, menstrual cycle frequency, age of first birth, duration of breastfeeding, etc.) [149] that have a direct effect on the duration of active estrogen effects on a woman organism and, accordingly, can modify the hormone-mediated BC-significant effects of SNP, controlling the level of SHBG in the organism. Nutritional characteristics are essential for BC-risk modification [149]. It is believed that ultra-processed foods (containing large amounts of sodium, fats, and sugar) contribute both to the development of obesity and increase the risk of developing BC [149]. It is indicated that an increase in the amount of ultra-processed foods in the diet by 10% leads to an increase in BC risk of 11% [150]. On the contrary, a diet high in vitamins, fiber, fruits, vegetables, legumes, etc., will help reduce both body weight and BC risk [151]. Studying the specific manifestations of the modifying effects of the above-mentioned BC-significant factors in subsequent genetic studies will allow for a better understanding of the mechanisms underlying these modifications, which will create conditions for their use in practice.

Thus, as a result of the study, new data were obtained on the risk role of SHBG-reducing SNP rs10454142 *PPP1R21* and the role of its absence on the occurrence of the disease in women without obesity. The data obtained in the future (after confirmation in other studies) can be used to form a high-risk group for BC among obese women (with the SHBG-reducing allele C rs10454142 *PPP1R21*), and in this group of women, weight loss (BMI) can be recommended as an effective preventive measure aimed at reducing the BC risk since, according to our data, among individuals without obesity, SHBG-reducing SNP rs10454142 *PPP1R21* was no longer associated with an increased risk of BC. 

As certain limitations of our study, the following should be noted: (a) the need for additional information (including experimental) on the differences in the functionality of SHBG-significant genetic determinants of BC in adipose tissue in obese and non-obese individuals; (b) determination of the level of a number of sex hormones associated with SHBG (estrogens, testosterone, etc., as well as SHBG itself), as the risk of BC in the studied groups of women (obese/non-obese) would allow for a more reasoned confirmation of the alleged pathophysiological mechanisms underlying the differences in the involvement of rs10454142 *PPP1R21* in the formation of the disease in obese/non-obese individuals; (c) certain limitations with the use of BMI as an indicator of the presence/absence of obesity (BMI ≥ 30/BMI < 30, respectively), which is based on a direct calculation taking into account height and weight and does not take into account the percentage of fat in the human body.

## 5. Conclusions

In our study, we detected an obesity-dependent association of the rs10454142 *PPP1R21* with BC in women. The SHBG-lowering allele C rs10454142 *PPP1R21* was a BC-risk factor in obese women and was not BC-linked in non-obese women.

## Figures and Tables

**Table 1:** Phenotypic characteristics of the study participants.

Parameters	BMI ≥ 30			BMI < 30		
	BC Patients $\bar{X}$ ± SD/%(n)	Controls $\bar{X}$ ± SD/%(n)	*p*	BC Patients $\bar{X}$ ± SD/%(n)	Controls $\bar{X}$ ± SD/%(n)	*p*
*N*	119	253	-	239	887	-
Age, years (min–max)	58.97 ± 10.67 (33–84)	58.01 ± 10.01 (31–80)	0.22	53.58 ± 13.12 (28–82)	52.77 ± 12.27 (29–80)	0.14
<50 years	26.89 (32)	27.67 (70)	0.98	37.24 (89)	40.70 (361)	0.37
≥50 years	73.11 (87)	72.73 (183)		62.76 (150)	59.30 (526)	
BMI, kg/m^2^	34.95 ± 4.76	33.12 ± 4.04	0.001	27.55 ± 2.85	26.13 ± 2.59	0.0004
Age at menarche, years	12.11 ± 1.02	12.30 ± 1.04	0.71	12.57 ± 1.05	12.78 ± 1.08	0.46
Age at menopause, years	48.58 ± 4.13	48.21 ± 4.01	0.65	48.08 ± 4.07	47.79 ± 4.02	0.36
Menstruation status						
Premenopausal	24.37 (29)	27.27 (69)	0.64	35.56 (85)	39.01 (346)	0.37
Postmenopausal	75.63 (90)	72.73 (184)		64.44 (154)	60.99 (541)	
Smoker (yes)	20.17 (24)	16.60 (42)	0.49	23.01 (55)	19.73 (175)	0.31
Biochemical parameters						
Fasting blood glucose (mmol/L)	8.76 ± 0.89	8.08 ± 0.75	<0.001	6.17 ± 0.75	5.19 ± 0.69	<0.001
TC (mmol/L)	6.34 ± 1.10	5.87 ± 1.02	<0.001	5.26 ± 1.01	4.76 ± 0.91	<0.001
HDL-C (mmol/L)	1.13 ± 0.45	1.26 ± 0.36	<0.001	1.40 ± 0.40	1.49 ± 0.42	<0.001
LDL-C (mmol/L)	4.31 ± 0.95	4.00 ± 0.86	<0.001	3.39 ± 0.79	3.07 ± 0.72	<0.001
TG (mmol/L)	1.98 ± 1.03	1.72 ± 1.00	<0.001	1.38 ± 0.64	1.21 ± 0.52	<0.001
Clinicopathological parameters of BC patients						
Stage of the cancer	T_0_–T_2_—79%, T_3_–T_4_—21%			T_0_–T_2_—72%, T_3_–T_4_—28%		
Lymph node involvement (N)	negative—50%, positive—50%			negative—46%, positive—54%		
Estrogen receptor (ER)	negative—31%, positive—69%			negative—36%, positive—64%		
Progesterone receptor (PR)	negative—38%, positive—62%			negative—43%, positive—57%		
Human epidermal growth factor receptor 2 (HER2)	negative—60%, positive—40%			negative—66%, positive—34%		
Tumor histological type	ductal—95%, lobular—5%			ductal—94%, lobular—6%		
Tumor histological grade (G)	G1/G2—70%, G3—30%			G1/G2—67%, G3—33%		
Progression	absent—68%, present—32%			absent—65%, present—35%		
Metastasis	absent—80%, present—20%			absent—77%, present—23%		
Death	absent—76%, present—24%			absent—83%, present—17%		

Note: G1, well differentiated; G2, moderately differentiated; G3, poorly differentiated.

**Table 2:** Associations of the studied gene polymorphisms with breast cancer among BMI < 30 and BMI ≥ 30 females.

Chr	SNP	Gene	Minor Allele	n	Allelic Model				Additive Model				Dominant Model				Recessive Model			
					OR	95%CI		*p*	OR	95%CI		*p*	OR	95%CI		*p*	OR	95%CI		*p*
						L95	U95			L95	U95			L95	U95			L95	U95	
Females with BMI < 30																				
1	rs17496332	*PRMT6*	G	1065	0.97	0.79	1.21	0.809	0.97	0.75	1.27	0.847	0.87	0.60	1.25	0.447	1.19	0.72	1.98	0.497
2	rs780093	*GCKR*	T	1087	1.00	0.81	1.23	0.987	0.87	0.67	1.14	0.314	0.85	0.58	1.25	0.407	0.81	0.49	1.34	0.415
2	rs10454142	*PPP1R21*	C	1072	1.02	0.82	1.28	0.837	1.15	0.87	1.53	0.331	1.13	0.78	1.65	0.508	1.38	0.75	2.53	0.301
7	rs3779195	*BAIAP2L1*	A	1073	1.05	0.81	1.36	0.726	1.12	0.80	1.56	0.522	1.18	0.80	1.75	0.405	0.89	0.32	2.51	0.827
8	rs440837	*ZBTB10*	G	1053	1.00	0.79	1.27	0.988	0.98	0.73	1.32	0.911	0.82	0.57	1.02	0.312	1.71	0.90	3.27	0.102
10	rs7910927	*JMJD1C*	T	1087	0.89	0.73	1.10	0.276	0.90	0.70	1.17	0.446	0.84	0.56	1.27	0.415	0.91	0.59	1.40	0.668
12	rs4149056	*SLCO1B1*	C	1041	0.88	0.69	1.14	0.336	1.03	0.75	1.42	0.838	1.08	0.73	1.58	0.709	0.88	0.36	2.14	0.779
15	rs8023580	*NR2F2*	C	1085	0.87	0.69	1.10	0.246	0.96	0.72	1.27	0.760	1.07	0.74	1.55	0.707	0.60	0.29	1.24	0.169
17	rs12150660	*SHBG*	T	1090	0.96	0.76	1.21	0.712	0.83	0.62	1.12	0.231	0.83	0.57	1.20	0.326	0.67	0.31	1.44	0.307
Females with BMI ≥ 30																				
1	rs17496332	*PRMT6*	G	357	0.86	0.62	1.19	0.362	1.02	0.69	1.50	0.919	0.93	0.55	1.58	0.787	1.27	0.59	2.76	0.542
2	rs780093	*GCKR*	T	358	0.86	0.63	1.19	0.371	0.91	0.62	1.34	0.635	1.03	0.60	1.77	0.920	0.65	0.29	1.44	0.289
2	rs10454142	*PPP1R21*	C	352	**1.52**	**1.10**	**2.11**	**0.012**	**1.73**	**1.15**	**2.62**	**0.009**	**1.95**	**1.13**	**3.37**	**0.016**	2.06	0.87	4.89	0.099
7	rs3779195	*BAIAP2L1*	A	348	1.15	0.75	1.76	0.514	0.98	0.60	1.61	0.949	1.09	0.61	1.94	0.768	0.43	0.08	2.45	0.345
8	rs440837	*ZBTB10*	G	355	0.79	0.54	1.17	0.235	0.76	0.48	1.19	0.230	0.72	0.42	1.23	0.230	0.70	0.19	2.57	0.594
10	rs7910927	*JMJD1C*	T	359	1.04	0.76	1.42	0.801	1.03	0.70	1.51	0.888	0.98	0.54	1.78	0.949	1.10	0.58	2.09	0.761
12	rs4149056	*SLCO1B1*	C	344	0.97	0.66	1.42	0.877	0.95	0.59	1.53	0.831	0.88	0.51	1.52	0.646	1.54	0.36	6.54	0.557
15	rs8023580	*NR2F2*	C	355	0.98	0.69	1.40	0.930	0.82	0.53	1.27	0.375	0.82	0.49	1.39	0.470	0.63	0.19	2.11	0.453
17	rs12150660	*SHBG*	T	362	1.11	0.77	1.58	0.583	1.16	0.77	1.76	0.480	1.10	0.65	1.85	0.728	1.72	0.63	4.69	0.288

Note: All results were obtained after adjustment for covariates; OR, odds ratio; 95% CI, 95% confidence interval. P_perm_ values < 0.025 are shown in bold.

**Table 3:** Possible functionality of the BC-correlated locus rs10454142 *PPP1R21* and proxy SNP (r ≥ 0.80) (in silico data).

**SNP** **(Position hg38)** **(r^2^, LD)**	Haploreg and GTE-Portal Data			GTE-Portal Data				Haploreg Data
	Liver	Adipocyte-Cultured Cells		Visceral Adipose		Subcutaneous Adipose		Transcription Factors
		Mesenchymal Stem-Cell-Derived Adipocyte-Cultured Cells	Adipose Nuclei	eQTL	sQTL	eQTL	sQTL	
rs17855177 (48375113) (r^2^ = 0.81, LD = 0.99)	*GTF2A1L*			*GTF2A1L*, *STON1-GTF2A1L*, *RP11-460M2.1*	*GTF2A1L*, *STON1*, *PPP1R21*, *STON1-GTF2A1L*	*GTF2A1L*, *STON1-GTF2A1L*, *PPP1R21*	*GTF2A1L*, *STON1*, *PPP1R21*	
rs78597273 (48380665) (r^2^ = 0.81, LD = 0.99)	*GTF2A1L*			*GTF2A1L*, *STON1-GTF2A1L*, *RP11-460M2.1*	*GTF2A1L*, *STON1*, *PPP1R21*, *STON1-GTF2A1L*	*GTF2A1L*, *STON1-GTF2A1L*, *PPP1R21*	*GTF2A1L*, *STON1*, *PPP1R21*	MIZF
rs11689645 (48381420) (r^2^ = 0.81, LD = 0.99)	*GTF2A1L*			*GTF2A1L*, *STON1-GTF2A1L*, *RP11-460M2.1*	*GTF2A1L*, *STON1*, *PPP1R21*, *STON1-GTF2A1L*	*GTF2A1L*, *STON1-GTF2A1L*	*GTF2A1L*, *STON1*, *PPP1R21*	AP-1, AP-2, BAF155, BATF, GR, Myc, BCL, Bach1, Bach2, GATA, HMGN3, KAP1, Maf, NF-E2, STAT, PRDM1, TCF4, p300
rs111960813 (48404376) (r^2^ = 0.80, LD = 0.93)	*GTF2A1L*			*GTF2A1L*, *STON1-GTF2A1L*, *RP11-460M2.1*	*GTF2A1L*, *STON1*, *PPP1R21*, *STON1-GTF2A1L*	*GTF2A1L*, *STON1-GTF2A1L*	*GTF2A1L*, *STON1*, *PPP1R21*	ELF1, Myc, ZBRK1
rs56391806 (48404838) (r^2^ = 0.85, LD = 0.98)	*GTF2A1L*			*GTF2A1L*, *STON1-GTF2A1L*, *RP11-460M2.1*	*GTF2A1L*, *STON1*, *PPP1R21*, *STON1-GTF2A1L*	*GTF2A1L*, *STON1-GTF2A1L*, *PPP1R21*	*GTF2A1L*, *STON1*, *PPP1R21*	Fox, Hoxb6
rs55744465 (48405316) (r^2^ = 0.85, LD = 0.98)	*GTF2A1L*			*GTF2A1L*, *STON1-GTF2A1L*, *RP11-460M2.1*	*GTF2A1L*, *STON1*, *PPP1R21*, *STON1-GTF2A1L*	*GTF2A1L*, *STON1-GTF2A1L*	*GTF2A1L*, *STON1*, *PPP1R21*	Hoxa5
rs201414717 (48419259) (r2 = 1.00, LD = 1.00)	* H3K4me1_Enh H3K4me3_Pro H3K27ac_Enh H3K9ac_Pro	H3K4me1_Enh H3K9ac_Pro	H3K4me1_Enh H3K27ac_Enh H3K9ac_Pro	*	*	*	*	AP-4, CACD, WT1, YY1, TAL1, TCF12, Rad21, LBP-1, ZNF219
**rs10454142** **(48419260)**	***GTF2A1L*** **H3K4me1_Enh** **H3K4me3_Pro H3K27ac_Enh H3K9ac_Pro**	**H3K4me1_Enh H3K9ac_Pro**	**H3K4me1_Enh** **H3K27ac_Enh H3K9ac_Pro**	***GTF2A1L*** **, ** ***STON1-GTF2A1L*, *RP11-460M2.1***	***GTF2A1L*** **, *STON1*, *PPP1R21*, *STON1-GTF2A1L***	***GTF2A1L*** **, ** ***STON1-GTF2A1L***	***GTF2A1L*** **, ** ***STON1*, ** ***PPP1R21***	**NF-kappaB**
rs10454143 (48419261) (r^2^ = 1.00, LD = 1.00)	*GTF2A1L* H3K4me1_Enh H3K4me3_Pro H3K27ac_Enh H3K9ac_Pro	H3K4me1_Enh H3K9ac_Pro	H3K4me1_Enh H3K27ac_Enh H3K9ac_Pro	*GTF2A1L*, *STON1-GTF2A1L*, *RP11-460M2.1*	*GTF2A1L*, *STON1*,*PPP1R21*, *STON1-GTF2A1L*	*GTF2A1L*, *STON1-GTF2A1L*	*GTF2A1L*, *STON1*, *PPP1R21*	Barx1, CEBPD, Hoxa3
rs13399936 (48426987) (r^2^ = 0.87, LD = 0.96)	*GTF2A1L*			*GTF2A1L*, *STON1-GTF2A1L*, *RP11-460M2.1*	*GTF2A1L*, *STON1*,*PPP1R21*, *STON1-GTF2A1L*	*GTF2A1L*, *STON1-GTF2A1L*	*GTF2A1L*, *STON1*, *PPP1R21*	
rs4638844 (48427445) (r^2^ = 0.81, LD = 0.94)	*			*	*	*	*	CIZ, FAC1, Foxa, Foxd3, Foxj2, Foxk1 Foxo, Foxp1, HDAC2, Irf, Pax-4, Sox, RREB-1, Zfp105, p300

Note: * The information in the GTE-portal database is not provided; H3K4me1_Enh, SNP location in the region of H3K4me1 histones marking enhancers; H3K27ac_Enh, active enhancers; H3K4me3_Pro, promoters; H3K9ac_Pro, active promoters. Bold text highlights BC-causal SNP.

**Table 4:** Prognostic potential of rs10454142 *PPP1R21* and proxy SNPs (r ≥ 0.80) as drivers of tumor development (regBase-CAN data).

SNP (Position hg38) (r^2^, LD)	Score	Phred Score	Potential Role
rs17855177 (48375113) (r^2^ = 0.81, LD = 0.99)	0.9983	22.4912	Likely cancer driver
rs78597273 (48380665) (r^2^ = 0.81, LD = 0.99)	0.8876	8.1965	Likely cancer driver
rs11689645 (48381420) (r^2^ = 0.81, LD = 0.99)	0.4049	4.4773	-
rs111960813 (48404376) (r^2^ = 0.80, LD = 0.93)	0.3714	4.2968	-
rs56391806 (48404838) (r^2^ = 0.85, LD = 0.98)	0.8828	8.1117	Likely cancer driver
rs55744465 (48405316) (r^2^ = 0.85, LD = 0.98)	0.7368	6.4687	Likely cancer driver
rs10454142 (48419260)	0.7992	7.0231	Likely cancer driver
rs10454143 (48419261) (r^2^ = 1.00, LD = 1.00)	0.7429	6.5174	Likely cancer driver
rs13399936 (48426987) (r^2^ = 0.87, LD = 0.96)	0.9669	10.8931	Likely cancer driver
rs4638844 (48427445) (r^2^ = 0.81, LD = 0.94)	0.9989	25.0004	Likely cancer driver

## Data Availability

The data generated in the present study are available from the corresponding author upon reasonable request.

## Supplementary Materials

The following supporting information can be downloaded at: https://www.mdpi.com/article/10.3390/biomedicines12040818/s1, Table S1: The GWAS data about associations of the studied candidate gene polymorphisms with the circulating SHBG and other sex hormone concentrations; Table S2: The allele and genotype frequencies of the studied SNPs in the breast cancer and control groups with BMI < 30; Table S3: The allele and genotype frequencies of the studied SNPs in the breast cancer and control groups with BMI ≥ 30; Table S4: Associations of BC-associated rs10454142 and strongly linked (r^2^>0.80) SNPs with it with the level of methylation of genome regions (mQTL) in various organs and tissues (according to QTLbase (http://www.mulinlab.org/qtlbase/index.html, accessed on, 20 August 2023); Table S5: Effect of rs10454142 *PPP1R21* and SNPs in high LD (r^2^ ≥ 0.80) on gene expression level (according to Genotype-Tissue Expression (GTEx) (http://www.gtexportal.org/, accessed on 10 August 2023)) (*p* < 8.0 × 10^−5^, FDR ≤ 0.05); Table S6: Effect of rs10454142 *PPP1R21* and SNPs in high LD (r^2^ ≥ 0.80) on alternative splicing level (according to Genotype-Tissue Expression (GTEx) (http://www.gtexportal.org/, accessed on 10 August 2023)) (*p* < 8.0 × 10^−5^, FDR ≤ 0.05); Table S7: Regulatory effects of the BC-associated SNP rs10454142 *PPP1R21* and SNPs in high LD (r^2^ ≥ 0.80) (HaploReg, v4.1, http://archive.broadinstitute.org/mammals/haploreg/haploreg.php, accessed on 5 August 2023).
