# Supplementary material for: Obesity-Dependent Association of the rs10454142 PPP1R21 with Breast Cancer
Source: Biomedicines. 2024 Apr 8;12(4):818. doi: 10.3390/biomedicines12040818 (PMC11048332; doi:10.3390/biomedicines12040818)
Supplement: Supplementary file 1 [file biomedicines-12-00818-s001.zip › +Table S1.pdf]

Supplementary Table 1. The GWAS data about associations of the studied candidate gene polymorphisms with the circulating SHBG and other sex hormone concentrations.

SNP, gene	Chromosome position (hg38)	Phenotype	Association (significance) (affected allele)	Reference
rs17496332 *PRMT6*	1p13.3 (107003753)	SHBG	β=-0.028 (p=1×10^{-11}) (A)	[19]
rs780093 *GCKR*	2p23.3 (27519736)	SHBG	β=-0.032 (p=2×10^{-16}) (T)	[19]
rs10454142 *FOXN2*	2p16.3 (48419260)	SHBG	β=0.026 (p=1×10^{-7}) (T)	[19]
rs3779195 *BAIAP2L1*	7q21.3 (98364050)	SHBG	β=-0.033 (p=3×10^{-8}) (A)	[19]
		SHBG (women, pre-menopause)	β=-2.41 (p=9×10^{-09}) (A)	[44]
rs440837 *ZBTB10*	8q21.13 (80549739)	SHBG	β=-0.030 (p=3×10^{-9}) (A)	[19]
		SHBG (women, post-menopause)	β=1.43 (p=1×10^{-12}) (G)	[44]
		SHBG (men)	β=0.57 (p=8×10^{-9}) (G)	[44]
rs7910927 *JMJD1C*	10q21.3 (63379150)	SHBG	β=-0.048 (p=6×10^{-35}) (T)	[19]
rs4149056 *SLCO1B1*	12p12.1 (21178615)	SHBG	β=0.029 (p=2×10^{-8}) (T)	[19]
		low testosterone levels (men)	OR=1.14 (p=7×10^{-16}) (C)	[45]
		testosterone (women)	β=0.028 (p=5×10^{-10}) (C)	[46]
		SHBG (women)	β=-0.065 (p =5×10^{-48}) (C)	[46]
		SHBG (women, pre-menopause)	β=-0.062 (p=8×10^{-11}) (C)	[46]
		SHBG (women, post-menopause)	β=-0.079 (p=7×10^{-34}) (C)	[46]
		bioavailable testosterone (women)	β=0.02 (p=2×10^{-16}) (C)	[44]
		SHBG (men)	β=-1.23 (p=7×10^{-29}) (C)	[44]
		SHBG (women)	β=0.030 (p=1×10^{-73}) (T)	[47]
		SHBG (men)	β=0.032 (p=6×10^{-99}) (T)	[47]
		total testosterone (women)	β=-0.029 (p=1×10^{-14}) (T)	[47]

		bioavailable testosterone (women)	β=-0.043 (p=3×10^{-35}) (T)	[47]
		total testosterone (men)	β=0.054 (p=1×10^{-39}) (T)	[47]
rs8023580 *PPP1R21*	15q26.2 (96165062)	SHBG	β=-0.03 (p=8×10^{-12}) (T)	[19]
		low testosterone levels (men)	OR=1.13 (p=1×10^{-19}) (T)	[45]
rs12150660 *SHBG*	17p13.1 (7618597)	SHBG	β=0.103 (p=2×10^{-106}) (T)	[19]
		SHBG (women)	β=6.14 (p=1×10^{-300}) (T)	[44]
		SHBG (men)	β=3.9 (p=2×10^{-75}) (T)	[48]
		total testosterone (men)	β=31.8 (p=1×10^{-41}) (T)	[48]
