# Supplementary material for: Obesity-Dependent Association of the rs10454142 PPP1R21 with Breast Cancer
Source: Biomedicines. 2024 Apr 8;12(4):818. doi: 10.3390/biomedicines12040818 (PMC11048332; doi:10.3390/biomedicines12040818)
Supplement: Supplementary file 1 [file biomedicines-12-00818-s001.zip › +Table S2.pdf]

Supplementary Table 2. The allele and genotype frequencies of the studied SNPs in the breast cancer and control groups with BMI<30.

Chr	SNP	Gene	Minor allele	Major allele	Minor allele frequency	Number of the studied chromosomes	Genotype distribution*	H_o	H_e	P_{HWE}
Breast cancer patients (n=239)										
1	rs17496332	*PRMT6*	G	A	0.360	470	37/95/103	0.40	0.46	0.066
2	rs780093	*GCKR*	T	C	0.405	472	35/121/80	0.51	0.48	0.348
2	rs10454142	*PPP1R21*	C	T	0.323	462	21/107/103	0.46	0.44	0.451
7	rs3779195	*BAIAP2L1*	A	T	0.190	464	7/74/151	0.32	0.31	0.673
8	rs440837	*ZBTB10*	G	A	0.246	464	18/78/136	0.34	0.37	0.158
10	rs7910927	*JMJD1C*	T	G	0.475	472	50/124/62	0.53	0.50	0.437
12	rs4149056	*SLCO1B1*	C	T	0.204	470	8/80/147	0.34	0.33	0.552
15	rs8023580	*NR2F2*	C	T	0.265	472	15/95/126	0.40	0.39	0.738
17	rs12150660	*SHBG*	T	G	0.246	472	14/88/134	0.37	0.37	1.000
Control group (n=887)										
1	rs17496332	*PRMT6*	G	A	0.366	1660	116/375/339	0.45	0.46	0.455
2	rs780093	*GCKR*	T	C	0.404	1702	141/406/304	0.48	0.48	0.776
2	rs10454142	*PPP1R21*	C	T	0.318	1682	88/358/395	0.43	0.43	0.633
7	rs3779195	*BAIAP2L1*	A	T	0.183	1682	35/237/569	0.28	0.30	0.106
8	rs440837	*ZBTB10*	G	A	0.246	1642	46/312/463	0.38	0.37	0.572
10	rs7910927	*JMJD1C*	T	G	0.503	1702	218/420/213	0.49	0.50	0.732
12	rs4149056	*SLCO1B1*	C	T	0.225	1612	47/269/490	0.33	0.35	0.226
15	rs8023580	*NR2F2*	C	T	0.292	1698	82/332/435	0.39	0.41	0.115
17	rs12150660	*SHBG*	T	G	0.254	1708	65/304/485	0.36	0.38	0.086

Note: * minor allele homozygotes / heterozygotes / major allele homozygotes
