# Supplementary material for: Obesity-Dependent Association of the rs10454142 PPP1R21 with Breast Cancer
Source: Biomedicines. 2024 Apr 8;12(4):818. doi: 10.3390/biomedicines12040818 (PMC11048332; doi:10.3390/biomedicines12040818)
Supplement: Supplementary file 1 [file biomedicines-12-00818-s001.zip › +Table S3.pdf]

Supplementary Table 3. The allele and genotype frequencies of the studied SNPs in the breast cancer and control groups with BMI≥30.

Chr	SNP	Gene	Minor allele	Major allele	Minor allele frequency	Number of the studied chromosomes	Genotype distribution*	H_o	H_e	P_{HWE}
Breast cancer patients (n=119)										
1	rs17496332	*PRMT6*	G	A	0.346	234	14/53/50	0.45	0.45	1.000
2	rs780093	*GCKR*	T	C	0.372	234	11/65/41	0.56	0.47	0.051
2	rs10454142	*PPP1R21*	C	T	0.397	234	15/63/39	0.54	0.48	0.246
7	rs3779195	*BAIAP2L1*	A	T	0.174	224	2/35/75	0.31	0.29	0.518
8	rs440837	*ZBTB10*	G	A	0.195	236	4/38/76	0.32	0.31	1.000
10	rs7910927	*JMJD1C*	T	G	0.483	236	26/62/30	0.53	0.50	0.712
12	rs4149056	*SLCO1B1*	C	T	0.216	236	5/41/72	0.35	0.34	1.000
15	rs8023580	*NR2F2*	C	T	0.263	236	5/52/61	0.44	0.39	0.162
17	rs12150660	*SHBG*	T	G	0.263	236	9/44/65	0.37	0.39	0.641
Control group (n=253)										
1	rs17496332	*PRMT6*	G	A	0.381	480	36/111/93	0.46	0.47	0.785
2	rs780093	*GCKR*	T	C	0.407	482	43/110/88	0.46	0.48	0.424
2	rs10454142	*PPP1R21*	C	T	0.302	470	19/104/112	0.44	0.42	0.536
7	rs3779195	*BAIAP2L1*	A	T	0.155	472	9/55/172	0.23	0.26	0.129
8	rs440837	*ZBTB10*	G	A	0.234	474	11/89/137	0.38	0.36	0.587
10	rs7910927	*JMJD1C*	T	G	0.473	482	47/134/60	0.56	0.50	0.093
12	rs4149056	*SLCO1B1*	C	T	0.221	452	7/86/133	0.38	0.34	0.174
15	rs8023580	*NR2F2*	C	T	0.266	474	19/88/130	0.37	0.39	0.505
17	rs12150660	*SHBG*	T	G	0.244	488	16/87/141	0.36	0.37	0.604

Note: * minor allele homozygotes / heterozygotes / major allele homozygotes
